# Multisystem Inflammatory Syndrome in Neonates Born to Mothers with SARS-CoV-2 Infection (MIS-N) and in Neonates and Infants Younger Than 6 Months with Acquired COVID-19 (MIS-C): A Systematic Review

**DOI:** 10.3390/v14040750

**Published:** 2022-04-02

**Authors:** Domenico Umberto De Rose, Flaminia Pugnaloni, Monica Calì, Sara Ronci, Stefano Caoci, Chiara Maddaloni, Ludovica Martini, Alessandra Santisi, Andrea Dotta, Cinzia Auriti

**Affiliations:** 1 Neonatal Intensive Care Unit, Medical and Surgical Department of Fetus—Newborn—Infant, “Bambino Gesù” Children’s Hospital IRCCS, 00165 Rome, Italy; domenico.derose@opbg.net (D.U.D.R.); flaminia.pugnaloni@opbg.net (F.P.); cali.monica.ct@gmail.com (M.C.); sararonci91@gmail.com (S.R.); stefano.caoci@opbg.net (S.C.); chiara.maddaloni@opbg.net (C.M.); ludovica.martini@opbg.net (L.M.); alessandra.santisi@opbg.net (A.S.); andrea.dotta@opbg.net (A.D.); 2 PhD Course in Microbiology, Immunology, Infectious Diseases, and Transplants (MIMIT), University of Rome “Tor Vergata”, 00133 Rome, Italy; 3 Pediatrics Department, “Sapienza” University, 00185 Rome, Italy

**Keywords:** neonate, SARS-CoV-2, COVID-19, infant, children, infection, autoantibodies, heart, respiratory distress

## Abstract

(1) Introduction: There is an increasing literature describing neonates born to mothers with SARS-CoV-2 infection (MIS-N) and infants infected with SARS-CoV-2 who presented with a severe disease (MIS-C). (2) Methods: To investigate clinical features of multisystem inflammatory syndrome in neonates and infants under six months of age, we used a systematic search to retrieve all relevant publications in the field. We screened in PubMed, EMBASE and Scopus for data published until 10 October 2021. (3) Results: Forty-eight articles were considered, including 29 case reports, six case series and 13 cohort studies. Regarding clinical features, only 18.2% of MIS-N neonates presented with fever; differently from older children with MIS-C, in which gastrointestinal symptoms were the most common manifestation, we displayed that cardiovascular dysfunction and respiratory distress are the prevalent findings both in neonates with MIS-N and in neonates/infants with MIS-C. (4) Conclusions: We suggest that all infants with suspected inflammatory disease should undergo echocardiography, due to the possibility of myocardial dysfunction and damage to the coronary arteries observed both in neonates with MIS-N and in neonates/infants with MIS-C. Moreover, we also summarize how they were treated and provide a therapeutic algorithm to suggest best management of these fragile infants.

## 1. Introduction

The severe acute respiratory syndrome coronavirus-2 (SARS-CoV-2) can infect all age groups. To date, children seem to have a more favourable clinical course of the related disease COVID-19, as observed during the last two years of pandemic [1]. Neonatal SARS-CoV-2 infections seem less frequent even now [2], probably due to the lower expression of SARS-CoV-2 entry receptors in nasal epithelium in both term and preterm neonates, compared with adults [3]. Neonatal cases are mostly linked to horizontal transmission due to familial clusters [4], although the rare possibility of a maternal fetal transmission has been demonstrated by Vivanti et al. [5].

However, there is an increasing literature describing SARS-CoV-2 infected children who become critically sick [6] because of the onset of a multisystem inflammatory syndrome named MIS-C. MIS-C has emerged as a significant COVID-19 related consequence, apparently not sparing neonates and infants, who can even require hospitalization and intensive care unit (ICU) support to survive [7,8]. This seems temporally associated with a SARS-CoV-2 infection.

**Figure 1:**
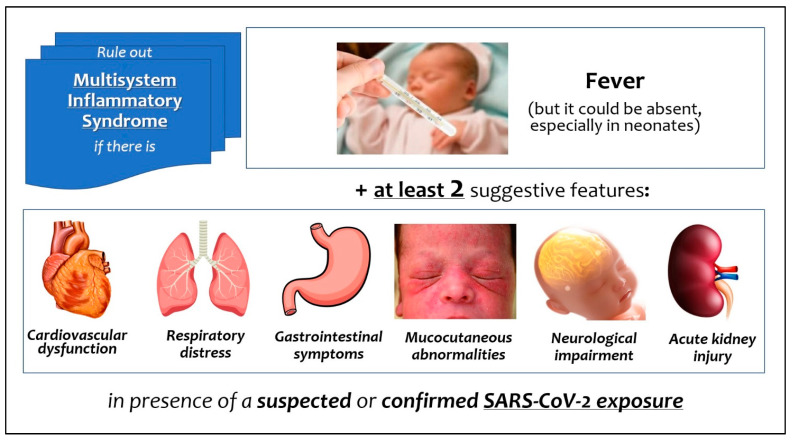
Signs and symptoms of multisystem inflammatory syndrome related to SARS-CoV-2 in neonates and infants.

The most frequent mode of onset is fever and multi-organ involvement (Figure 1), associated with a rise in inflammatory biomarkers [9]. A similar multisystem inflammatory syndrome has been documented in adults (MIS-A) [10]. Patients with MIS-C are mostly children older than 7 years [7], but Pawar et al. [11] were the first to distinguish the early neonatal inflammatory syndrome (with onset within one week of life) in infants born to mothers with COVID-19 contracted in pregnancy (MIS-N), from that complicating the neonatal infection contracted after birth [11].

In this review we conducted a thorough analysis and synthesis of previously described cases of MIS-N and MIS-C in neonates and infants, aiming to describe clinical features of multisystem inflammatory syndrome in these delicate patients. Moreover, we also summarize how these infants were treated, in order to provide preliminary suggestions for management in this new clinical scenario.

## 2. Materials and Methods

We performed this systematic review following Preferred Reporting Items for Systematic Reviews and Meta-Analyses (PRISMA) guidelines throughout the whole project. Prior to commencing the search, a detailed protocol was agreed to determine search modalities, eligibility criteria, and all methodological details. We searched for cohort, cross-sectional and case-control studies, as well as case series or case reports published as articles or letters to the editors describing neonates with multisystem inflammatory syndrome following maternal SARS-CoV-2 infection (MIS-N) or neonates and infants within first six months of life infected with SARS-CoV-2 and with MIS-C features.

We conducted an extensive search of the following databases (accessed on 10 October 2021): PubMed (https://pubmed.ncbi.nlm.nih.gov/), Scopus (https://www.scopus.com/search/form.uri?display=basic#basic) and Embase (https://www.embase.com/?phase=continueToApp#search). We used the following terms: ((“pediatric” AND “multisystem” AND “inflammatory” AND “syndrome”) OR (“multisystem” AND “inflammatory” AND “syndrome” AND “in” AND “children”)) AND “SARS-CoV-2”. Additional studies were identified by authors based on their knowledge in the field, if not already included by literature search. We excluded (1) all retrieved articles written in non-English language; (2) articles which did not clearly report the number of infants under six months of age.

Articles were assessed by independent researchers (DUDR, FP, MC, SR, SC, CM, LM and AS). Investigators evaluated abstracts and (where necessary) the full text of each article, excluding those not meeting the eligibility criteria, and removing duplicates. The CARE (Consensus-based Clinical Case Reporting Guideline Development) recommendations, specifically dedicated to case reports and series, were followed during the evaluation process. If an article was eligible but reported data on neonates and infants mixed with those on older children, eligible data were directly extracted. If there were uncertainties, they were resolved by discussion between the independent researchers and, if no agreement was reached, with the senior researcher (CA). We developed a dedicated online data extraction sheet (Excel 16: Microsoft Corporation, Redmond, WA, USA). Data from included records were independently extracted by each investigator using this data extraction sheet and then cross-verified. 

Since we expected the majority of analyzed articles to be case reports or case series, we used the Mayo Evidence-Based Practice Centre tool that is specifically dedicated to the evaluation of case report/series quality [12]. Two investigators (DUDR and CA) independently summarized the results of this evaluation by aggregating the eight binary responses into a 0–8 score. Evaluation results were also qualitatively summarized (Low-Intermediate-Good), as recommended by the tool creators. If discrepancies or uncertainties persisted, they were resolved by discussion between the two researchers (DUDR and CA). We performed calculations and statistics with Excel 16 (Microsoft Corporation, Redmond, WA, USA), reporting continuous data as median (interquartile range, IQR), and categorical data as numbers and percentages.

## 3. Results

### 3.1. Workflow of Review and Synthesis

**Figure 2:**
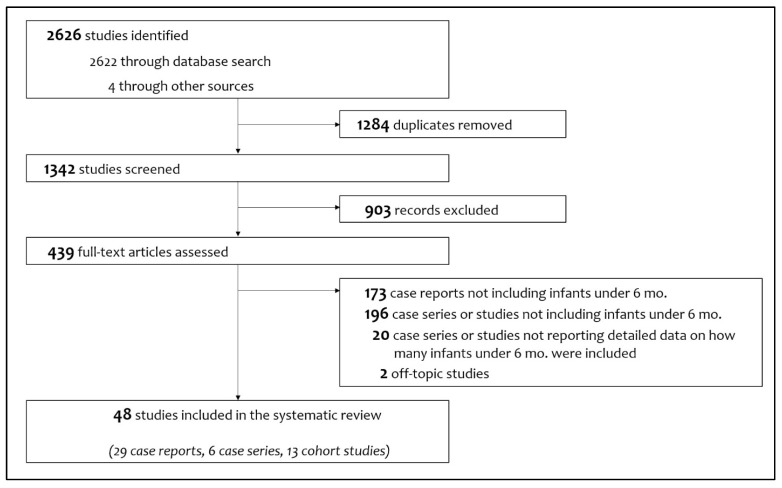
Flowchart of study selection process.

Figure 2 shows the study flow chart with included and excluded items (and the reasons for their exclusions). Finally, 48 articles were considered, consisting of 29 case reports [5,13,14,15,16,17,18,19,20,21,22,23,24,25,26,27,28,29,30,31,32,33,34,35,36,37,38,39,40], six case series [11,41,42,43,44,45] and 13 cohort studies [34,46,47,48,49,50,51,52,53,54,55,56,57].

We describe in Table 1 the characteristics of the included papers: we assessed as intermediate (median score 5 (5,6)) the methodological quality of case reports, case series and cohort studies which provided supplemental data with case descriptions. Ninety cases of neonates and infants under 6 months of age with multisystem inflammatory syndrome were described in the literature at the time of the search.

### 3.2. Neonates with MIS-N

Thirteen included papers [5,11,14,15,19,20,25,26,29,30,33,38,39] fully described 33 cases of neonates with multisystem inflammatory syndrome in the first week after birth, born to mothers with SARS-CoV-2 infection (Table 2). Twenty-four (72.7%) of these neonates, were born preterm, with a median gestational age of 34 [33,34,35,36] weeks and a median birthweight of 2020 [1890–2620] grams. The diagnosis was obtained with a median age of 2 (0–3) days. All infants were admitted to Neonatal Intensive Care Unit (NICU) and 17 (51.5%) of them required mechanical ventilation.

Prior maternal SARS-CoV-2 exposure occurred in all infants. Neonatal RT-PCR for SARS-CoV-2 resulted positive only in four neonates (12.1%), whereas a positive serology yielded a maternal perinatal SARS-CoV-2 infection in 25 cases (75.8%). 

Fever was observed only in six neonates (18.2%). Organ system involvement included cardiovascular dysfunction in 26 neonates (78.8%), respiratory distress in 22 (66.7%), gastrointestinal symptoms in nine (27.3%), mucocutaneous abnormalities in nine (27.3%), neurological impairment in eight (24.2%) and acute kidney injury in five (15.2%). None presented with musculoskeletal anomalies.

Laboratory tests showed mostly an increase in C-reactive protein (60.6%) and procalcitonin (27.3%), raised brain natriuretic peptide (NT-proBNP, 51.5%) and troponin (24.2%), increased D-dimer (84.4%) and interleukin-6 (18.2%), thrombocytopenia (18.2%), metabolic acidosis (15.1%), prolonged prothrombin time (6.1%), and hypoalbuminemia (9.1%).

Chest X-ray or chest CT discovered a pulmonary involvement in 12 neonates (36.4%), whereas echocardiography showed depressed ventricular functions and coronary anomalies in 17 (51.5%). 

Twenty-seven neonates (81.8%) received intravenous immunoglobulins (IVIG: a total of 2 g/kg splitted in two infusions of 1 g/kg/day, without a further dose) and intravenous steroids (mostly methylprednisolone). Eighteen neonates (54.5%) needed inotropic support. None was treated with biologic medications or antivirals. The use of large-spectrum antibiotics was described in 10/13 cases (76.9%). Acetylsalicylic acid was given to 6 neonates (18.2%) while thromboprophylaxis was used in 13 cases (39.4%). 

The median length of stay was 16 [13,14,15,16,17,18,19,20,21,22] days. The outcome was favourable in 30 neonates (90.9%); of these, a full-term male without comorbidities survived but required amputation of the right leg (because of an acute thrombosis of abdominal aorta) and a late preterm survived after peritoneal dialysis. Only two neonates died (the first on day 8 due to a multi-organ dysfunction and the second on day 11 because of necrotizing enterocolitis).

### 3.3. Neonates and Infants with MIS-C

Twenty-five included papers [13,16,17,18,21,22,23,24,27,28,31,32,34,35,36,37,40,41,42,43,44,45,47,52,53] describe 32 neonates and infants under the age of six months with multisystem inflammatory syndrome after the acquired SARS-CoV-2 infection (Table 3). Only six (18,8%) of these infants, had comorbidities. Thirteen infants (40.6%) required ICU admission, of whom 9/13 (69.2%) undergoing mechanical ventilation. Twenty-one infants (65.6%) had a positive RT-PCR for SARS-CoV-2, whereas in 12 (37.5%) previous exposure to SARS-CoV-2 was confirmed by a positive serology. In most cases, MIS-C was related to the positivity for SARS-CoV-2 of a family member in previous days or weeks (where reported): only one infant, hospitalized twice because of a late-onset sepsis, might have contracted the virus as a nosocomial infection.

Twenty-seven infants (84.4%) presented with fever. Organ system involvement included cardiovascular dysfunctions in 26 infants (81.3%), respiratory distress in 23 (71.9%), gastrointestinal symptoms in 17 (53.1%), neurological impairment in 15 (46.9%), mucocutaneous abnormalities in 14 (43.8%) and acute kidney injury in three (9.4%). Only one (3.1%) presented with fatigue as musculoskeletal anomaly. Laboratory tests showed increase in C-reactive protein (68.8%) more than in procalcitonin (18.8%), elevated ferritin (56.2%), raised brain natriuretic peptide (NT-proBNP, 40.6%) and troponin (34.3%), increased D-dimer (46.9%) and interleukin-6 (21.8%), hypoalbuminemia (34.3%), and thrombocytopenia (15.7%). 

Eight infants (25%) presented pulmonary involvement by chest X-ray and/or CT scan, whereas depressed ventricular function and coronaries anomalies were detected by echocardiography in 21 (65.6%). 

Twenty-two infants (68.8%) received intravenous immunoglobulins (IVIG: a total of 2 g/kg splitted in two infusions of 1 g/kg/day, without a further dose) and fifteen (46.9%) intravenous steroids (mostly methylprednisolone). Five infants (15.6%) needed inotropic neonatal support. Nine infants (28.1%) received biologic medications (five anakinra, one anakinra and then infliximab, one infliximab, one tocilizumab, one inhaled interferon α-1b). Three infants (9.4%) were treated with remdesivir as antiviral.

The use of large-spectrum antibiotics was described in 17 cases (53.1%). Acetylsalicylic acid was given to 14 infants (43.8%); thromboprophylaxis was used in 14 cases (43.8%) and warfarin was given to one infant (3.1%). 

The median length of stay was 15 (10–24) days. The outcome was favourable in 29 infants (90.6%), whereas four infants (12.5%) died: two neonate females within one month of life without comorbidities, a 2-months-old male with a familial hemophagocytic lymphohistiocytosis secondary to Griscelli syndrome type 2, and a 3-months-old baby with isolated coronary artery disease.

Analyzing data of neonates separately, among infants with MIS-C, only five (15.6%) had less than a month or before reaching term age if preterm born [18,22,37,39,46]. Three of five (60%) had fever. All had a cardiovascular and respiratory involvement, whereas two (40%) had gastrointestinal symptoms. Three (60%) received IVIG and four (80%) steroids. All required ICU admission and mechanical ventilation. Only two neonates (40%) died. 

### 3.4. Differences between Neonates with MIS-N and Neonates with MIS-C

When SARS-CoV-2 infection was acquired postnatally, fever was a frequent feature (60%). rather than in neonates with MIS-N (18.2%). All neonates had a severe course, requiring NICU admission. Organ system involvement was similar for both neonate groups, with prevalent cardiovascular and respiratory signs and symptoms. We found no significant differences between the two groups for laboratory tests and treatments. 

Two neonates died in both groups, with a slightly higher incidence in the MIS-C group although not significantly (2/33 MIS-N neonates versus 2/5 MIS-C neonates, *p* = 0.07). 

### 3.5. Incidence of Multisystem Inflammatory Syndrome within Six Months of Age

Twelve cohort studies [44,46,47,48,49,50,51,54,55,56,57,58], described 1063 children with multisystem inflammatory syndrome, included 31 infants within six months of age (Table 4). These data allow calculation that 2.9% of reported cases are related to infants younger than six months of age: of these, 10 were neonates (32.3%) and 21 were infants (67.7%).

## 4. Discussion

This systematic review is the first to synthesize the current data regarding MIS-N in neonates and MIS-C in neonates and infants younger than six months of age. The literature relating to multisystem inflammatory syndrome in these patients is mainly centred on case reports or small case series: we summarized the most frequent clinical features to describe this syndrome in neonates and provide some therapeutic suggestions. 

**Figure 3:**
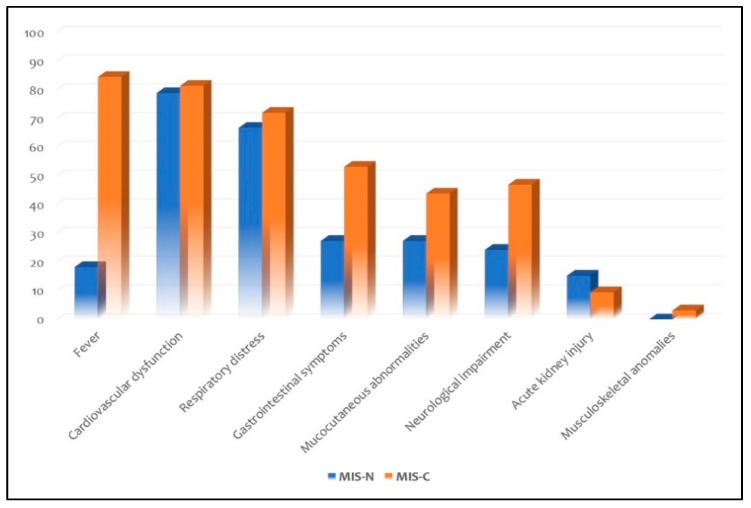
Distribution of clinical features in the subgroups of MIS-N and MIS-C.

Regarding clinical features, fever is a milestone in older children with MIS-C (99.3%) [59]: we confirmed this trend in young infants with MIS-C (84.4%). Conversely, only the 18.2% of neonates with MIS-N presented with fever, but temperature changes are not a constant finding in the onset of infectious and even in the inflammatory febrile pathologies in preterm and term neonates [60,61]. Therefore, differences in age of MIS-N and MIS-C infants could explain this disparity in fever incidence. Differently from older children with MIS-C, in which gastrointestinal symptoms were the most common manifestation (87.3%) [62], we demonstrated that cardiovascular dysfunction and respiratory distress are the prevalent findings both in neonates with MIS-N and in neonates/infants with MIS-C (Figure 3).

**Figure 4:**
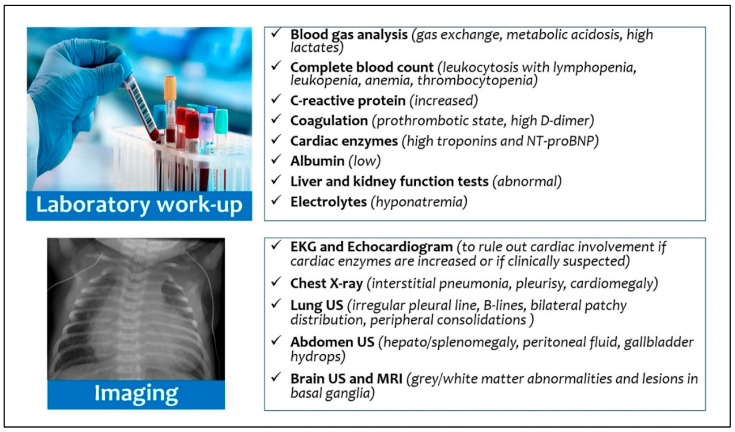
Diagnostic work-up for multisystem inflammatory syndrome in neonates and infants.

From the diagnostic point of view (Figure 4), although the current diagnostic reference standard for neonatal respiratory distress includes chest X-ray [63], only a third of patients with MIS-N or MIS-C showed pathologic chest X-ray. Recently, Musolino et al. suggested performing lung ultrasound (LUS) in patients with clinical suspicion of MIS-C, or without a certain diagnosis: the finding of many B-lines and pleural effusion would support the diagnosis of a systemic inflammatory disease [64]. Moreover, LUS can also reduce X-ray exposure, but its efficacy as diagnostic tool has been described only in a 6-months-old infant with MIS-C: the authors found an irregular pleural line, B-lines, with bilateral patchy distribution, and small peripheral consolidations [38].

All infants with suspected inflammatory disease should undergo echocardiography, due to the possibility of myocardial dysfunction and damage to the coronary arteries observed both in neonates with MIS-N [11,19,25,26,29,33,38,39] and in neonates/infants with MIS-C [13,18,22,24,28,32,35,36,37,40,41,42,43,45,47,52,53]. Indeed, functional echocardiography can provide a direct bed-side assessment of cardiovascular anomalies and hemodynamics [65]. We suggest performing echocardiography in neonates with MIS-N after 24–48 h of life (or previously in case of symptoms), considering hemodynamic changes that physiologically occur during transition from fetal to neonatal life, while in MIS-C infants echocardiography should be carried on admission. Furthermore, neonates with MIS-N had a higher need of inotropic support (54.5%) than infants with MIS-C (15.6%); targeted echocardiography also offers the advantage of longitudinally assessing infants and their response to therapeutic intervention [66]. The increase in cardiac enzymes (troponin) and cardiac function-related proteins (NT-proBNP) must induce a strong suspicion of myocardial involvement and requires careful monitoring [67]. Additionally, the presence of microvascular dysfunction has recently been characterized in pediatric patients with SARS-CoV-2 pneumonia [68]. 

Inflammatory markers could be also raised, but we identified that neonates with MIS-N had lower levels than neonates/infants with MIS-C. Similarly, Zhao et al. assessed that younger children with MIS-C had lower levels of inflammatory markers when compared to middle-age children and adolescents with MIS-C [69].

**Figure 5:**
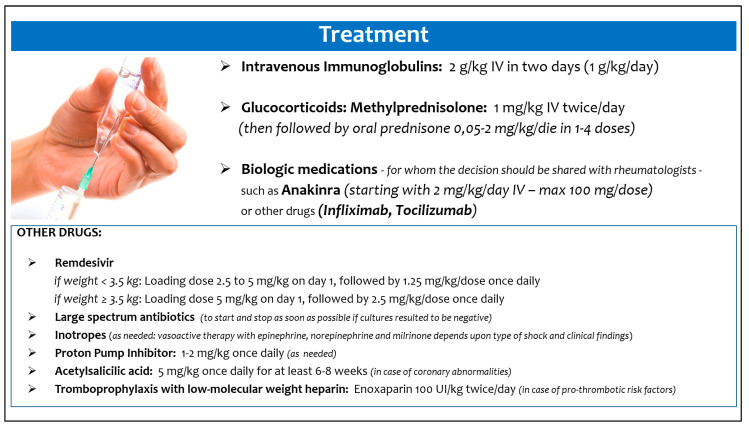
Suggested treatment of multisystem inflammatory syndrome in neonates and infants.

Moreover, we also tried to summarize how these infants were treated, in order to provide preliminary suggestions in management of this new clinical scenario (Figure 5), although data did not come from randomized studies [70]. The best treatment approach and the appropriate timing should be defined on an individual basis, as suggested by Cattalini et al. [71].

MIS-C and MIS-N seem to originate from immune-mediated mechanisms, in the setting of a suspected or confirmed SARS-CoV-2 infection: however, in MIS-N the primary source is the maternal infection in pregnancy, with transplacental passage of maternal antibodies [62], whereas in MIS-C the infant contracts a postnatal infection and mounts an antibody response with intact neutralization capability [72]. The efficacy of intravenous immunoglobulins (IVIG) in MIS-C, a common approach to activating inhibitory Fc-receptors and preventing membrane-attack complexes by complement factors, thereby mitigating autoantibody-mediated pathology [73], lends support to the hypothesis that autoantibodies contribute to MIS-C pathogenesis [74].

The majority of infants described in the literature received IVIG [11,13,14,19,20,24,25,27,28,34,35,37,38,39,40,42,43,44,45,47,52,53] and steroids (mostly methylprednisolone) [11,14,15,18,22,25,26,28,31,32,34,36,37,38,39,40,41,42,44,45,52]. According to results of a French study, the treatment with IVIG and methylprednisolone vs IVIG alone was associated with a more favourable course among children with MIS-C [18,22,45]. However, data interpretation is limited by the observational design of the study [75].

Contrarily, antivirals (such as remdesivir) were poorly used (in none of the neonates with MIS-N and only 9.4% of infants with MIS-C) [18,22,45]. Similarly, biologic medications were not given to neonates with MIS-N, whereas about a third of infants with MIS-C required treatment with anakinra, infliximab, tocilizumab, and inhaled interferon α-1b [16,34,36,40,44,45,47].

Most neonates and infants were also treated with large-spectrum antibiotics, given the overlapping clinical signs and symptoms with those of sepsis. Recently, Yock-Corrales and colleagues set the alarm regarding the high rate of antibiotic prescriptions (24.5%) in children with COVID-19 (in particular in those with more severe forms) [76]. Considering the high need for ICU admission in neonates with MIS-N (100%) and infants with MIS-C (40.6%) described in the included studies of this review, and given that an antibiotic treatment is often guaranteed in younger infants, the length of antibiotic therapy should be carefully evaluated: reducing patient exposure to large-spectrum antibiotics can result in avoiding the spread of multi-drug resistant organisms [77]. The time to positivity of blood cultures performed on admission could guide decisions on antibiotics administration in neonates, because most bacterial pathogens grow within 48 h [78,79]. 

Although the outcome was favourable in the majority of cases, the observed mortality was 9.2% in neonates with MIS-N and infants younger than under six months with MIS-C. Conversely, the reported mortality rate was 1.9% when all pediatric MIS-C cases reported in the literature were considered [59]. This is an expected finding, considering the higher cardiovascular and respiratory involvement in younger infants.

This study had several limitations. First, the available studies were mostly case reports or case series, with possible selection biases. Second, data on some variables were not accessible in all papers or were not uniformly reported. Third, there is no consensus about diagnostic criteria in MIS-C, whereas recently Pawar et al. proposed modified criteria for MIS-N [11]. Fourth, most of these data have been obtained in the “pre-Omicron” period. Indeed, since late November 2021, with the emergence of the new SARS-CoV-2 variant B.1.1.529 (named Omicron), the number of COVID-19 cases increased substantially. Although the symptoms of the new cases are reported to be mild to moderate [80], we do not know what the actual impact will be in younger infants, for whom a vaccine is not yet available.

## 5. Conclusions

Multisystem inflammatory syndrome, named MIS-N or MIS-C, related to SARS-CoV-2 exposure can occur in a high percentage of neonates and infants. In affected neonates common findings are cardiac dysfunction and the coronary artery dilation or aneurysms; thus, a complete echocardiography is strongly recommended in the diagnostic approach.

The studies that we have consulted reported an overall good prognosis, despite the frequent need of NICU and ICU admissions. 

Further epidemiological, clinical, immunological, and neurodevelopmental studies are needed to better clarify short- and long-term outcomes of neonates and infants with this inflammatory condition related to SARS-CoV-2 exposure.

## Figures and Tables

**Table 1:** Characteristics of articles included in the systematic review. Methodological quality of case description was assessed only for case report and case series, or cohort studies which provided supplemental data with case descriptions.

First Author	Article Type	Country	Quality Score	Overall Quality	Neonates and Infants < 6 Months with MIS (*n*)
Abdel-Haq [46]	Cohort study	United States	N.A.	N.A.	1
Acharyya [13]	Case report	India	4	Intermediate	1
Agrawal [14]	Case report	India	6	Good	1
Alharbi [47]	Cohort study	Saudi Arabia	5	Intermediate	2
Amonkar [15]	Case report	India	5	Intermediate	1
Antúnez-Montes [48]	Cohort study	Mexico	N.A.	N.A.	3
Caro-Dominguez [49]	Cohort study	Spain	N.A.	N.A.	1
Chandran [50]	Cohort study	India	N.A.	N.A.	3
Cui [16]	Case report	China	6	Good	1
Del Barba [17]	Case report	Italy	5	Intermediate	1
Diggikar [18]	Case report	India	5	Intermediate	1
Divekar [19]	Case report	United States	5	Intermediate	1
Diwakar [20]	Case report	India	5	Intermediate	1
Dugue [21]	Case report	United States	3	Intermediate	1
Dufort [51]	Cohort study	United States	N.A.	N.A.	1
Esteve-Sole [52]	Cohort study	Spain	3	Intermediate	1
Falah [53]	Cohort study	Pakistan	3	Intermediate	2
Frauenfelder [22]	Case report	United Kingdom	6	Good	1
García-Howard [23]	Case report	Spain	5	Intermediate	1
Giacomet [24]	Case report	Italy	5	Intermediate	1
Godfred-Cato [54]	Cohort study	United States	N.A.	N.A.	1
Grewal [55]	Cohort study	United States	N.A.	N.A.	9
Güllü [56]	Cohort study	Turkey	N.A.	N.A.	3
Kappanyil [25]	Case report	India	6	Good	1
Khaund Borkotoky [26]	Case report	India	5	Intermediate	1
Jones [27]	Case report	United States	5	Intermediate	1
Lad [28]	Case report	India	2	Low	1
Lima [29]	Case report	Brazil	6	Good	1
Lorenz [30]	Case report	Germany	3	Intermediate	1
Luna Santiago [31]	Case report	Mexico	3	Intermediate	1
Malle [41]	Case series	United States	6	Good	1
Mariani [32]	Case report	United States	3	Intermediate	1
Marino [42]	Case series	Italy	4	Intermediate	1
McCarty [33]	Case report	United States	5	Intermediate	1
Mehra [57]	Cohort study	India	N.A.	N.A.	1
Niño-Taravilla [58]	Cohort study	Chile	2	N.A.	2
Orlanski-Meyer [34]	Case report	Israel	5	Intermediate	1
Pawar [11]	Case series	India	6	Good	20
Rakha [43]	Case series	Egypt	5	Intermediate	5
Raut [35]	Case report	India	5	Intermediate	1
Richardson [40]	Case report	United Kingdom	6	Good	1
Rodriguez-Gonzalez [36]	Case report	Spain	5	Intermediate	1
Saha [37]	Case report	India	6	Good	1
Schoenmakers [38]	Case report	Netherlands	6	Good	1
Shaiba [39]	Case report	Saudi Arabia	6	Good	2
Shaiba [44]	Case series	Saudi Arabia	5	Intermediate	2
Villacis-Nunez [45]	Case series	United States	6	Good	1
Vivanti [5]	Case report	France	6	Good	1
Total of included infants					90

N.A. Not available.

**Table 2:** Characteristics of neonates with MIS-N with fully described cases.

Subject Number	First Author	Age/ Sex/ Birthweight/GA	Neonatal RT-PCR and Serology for SARS-CoV-2	Prior Maternal SARS-CoV-2 Exposure	Fever	Organ Involvement	Laboratory Work-Up	Imaging	Treatment	NICU Admission/Need for MV/Length of Hospital Stay	Outcome
1	Agrawal [14]	44 h/M/3300 g/39 w	RT-PCR neg/IgG pos/IgM neg	YES—positive maternal IgG (without vaccination): she had a history of contact with COVID-19 4 weeks before delivery but remained asymptomatic.	Yes	Hypotension; respiratory distress requiring MV; indurated ulcer with erythema noted on the occiput; non-bilious vomiting and abdominal distension—surgical abdomen	Leukocytosis (23,940), metabolic acidosis (pH 7.18), PCT 10.76, NT-proBNP 4297, D-dimer 1331	Normal inflation with minimal pulmonary infiltrates bilaterally (CXR); normal echocardiogram; dilated small and large intestines with absence of rectal gas and no radiological evidence of intestinal atresia or NEC (AXR)	IVIG, steroids, inotropes (dopamine), enoxaparin, aspirin	Yes/Yes/16 days	Favorable
2	Amonkar [15]	6 days/M/2400 g/full-term	RT-PCR neg/IgG pos/IgM pos	YES—maternal asymptomatic infection with positive IgG and IgM on day 11 of life in both mother and neonate	No	Respiratory distress requiring ventilatory support (not specified); progressive blackish discoloration of the toes of the right lower limb; irritability	Leukocytosis (17,100), CRP 18.6, PCT 1.28, albumin 3, NT-proBNP 12,194, ferritin 515, D-dimer 4110, IL-6 20.29	Acute thrombosis of abdominal aorta below renal arteries (80–90% occlusion at echocardiogram)	Steroids, r-TPA and surgical embolectomy, aspirin after limb amputation, unfractionated heparin	Yes/No/28 days	Favorable but he required amputation of the leg
3	Divekar [19]	At birth/F/1300 g/30 w	RT-PCR neg/IgG pos/IgM neg	YES—positive maternal RT-PCR prior to delivery	No	Hypotension and inadequate urine output; hypoxic respiratory failure requiring surfactant and HFOV; generalized anasarca; oliguric renal failure	Tn 189, NT-proBNP >5000, IL-6 21.9	Cardiomegaly and pulmonary edema (CXR); small pericardial effusion without tamponade, pathological coronary artery dilation and ventricular systolic dysfunction (echocardiogram)	IVIG	Yes/Yes/2 months	Favorable
4	Diwakar [20]	19 days/M/3910 g/39 w	RT-PCR neg/Positive IgG	YES—maternal fever a week before delivery; she resulted positive when the baby was admitted	Yes	Rash over forehead and cheeks on day 2; diarrhea	Thrombocytopenia (97 × 10^9^/L), transient neutropenia, CRP 12.13, hyponatremia (127), albumin 2.92, D-dimer 710, IL-6 1624	Clear lung fields (CXR); normal structure and function with normal coronary arteries (echocardiogram)	IVIG, aspirin	Yes/No/6 days	Favorable
5	Kappanayil [25]	24 days/F/3750 g/term	RT-PCR neg/IgG pos/IgM neg	YES—mother with a history of positive RT-PCR, with mild COVID-19 at 31 weeks GA, which was managed with symptomatic and supportive measures.	No	Hypotension and tachycardia; hypoxic respiratory failure; cool peripheries with erythema over the occiput and at bilateral gluteal regions; drowsiness	CRP 0.65, hyper-transamin-asemia (ALT 866 U/L and AST 2240 U/L), metabolic acidosis (pH 7.13), Tn 123, NT-proBNP 157,000, ferritin 56,400, D-dimer 20,000	Cardiomegaly (CXR); severe biventricular dysfunction; coronary arteries with normal luminal dimensions, but appeared prominent and hyper-choic (echocardiogram)	IVIG, steroids, inotropes (epinephrine and milrinone), furosemide, vitamin C and D, zinc, heparin	Yes/Yes/29 days	Favorable
6	Khaund Borkotoky [26]	4 h/M/4840 g/38 w	RT-PCR neg/IgG pos/IgM neg	YES—maternal fever and cough 3 weeks before delivery. On the day of delivery, her throat and nasal swab for COVID-19 PCR were negative. Anti-SARS-CoV-2 IgG were positive.	Yes	PPHN; hypoxic respiratory failure at 4 h of life and again on day 7; vasculitic rash on day 14; features suggestive of early NEC on day 14	Leukocytosis (19,800), thrombocytopenia (70 × 10^9^/L), CRP 3.91, albumin 2.2, Tn 171.2, NT-proBNP 6125, ferritin 1432, D-dimer >10,000, IL-6 43.49	Bilateral haze (CXR) and bilateral ground opacities considered consistent with COVID-19 (chest CD); PPHN (echocardiogram)	Steroids, sildenafil, inotropes (dopamine), furosemide	Yes/Yes/34 days	Favorable
7	Lima [29]	3 days/F/2400 g/33 w	RT-PCR pos/IgG pos/IgM neg	YES—flu-like symptoms at 29 weeks GA. At 32 weeks, the fetal echocardiogram revealed significant pericardial effusion with dilation of the vena cava and an overload in the cardiovascular system	No	Pericardial effusion; hemodynamic instability or bradycardia; respiratory distress and apnea requiring mechanical ventilation	CRP 1.1, metabolic acidosis (pH 7.12), Tn 38,000, ferritin 358, INR 1.4, D-dimer 2200, IL-6 56.4 in serum and 202.9 in pericardial fluid	Inflammatory ground glass pattern affecting less than 25% of the lung parenchyma (chest CT); pericardial effusion (echocardiogram)	Pericardiocentesis; red blood cell concentrate transfusion, inotropes (not specified amine)	Yes/Yes/23 days	Favorable
8	Lorenz [30]	24 h/F/NA/40 w	RT-PCR pos/NA	YES—mother with mild respiratory infection and loss of smell and taste, and fever during delivery (38 °C)	No	Respiratory distress at about 80 h of life, requiring CPAP and oxygen therapy until day 6 of life; encephalitic symptoms (lethargic but severely hyperexcitable, high pitched crying) at 54 h of life	NA	Bilateral viral pneumonia (CXR)	Paracetamol and caffeine	Yes/No/NA	Favorable
9	McCarty [33]	1st day/M/NA/34 w	RT-PCR neg/Serology NA	YES—mother with COVID-19 symptoms and positive RT-PCR	Yes	Severe pulmonary hypertension; respiratory distress requiring surfactant, mechanical ventilation and inhaled nitric oxide	Lymphopenia (230 at 48 h of life), thrombocytopenia (25 × 10^9^/L), CRP 6.78, metabolic acidosis (pH 7.00)	Diffuse bilateral granular opacities (CXR); severe PPHN (echocardiogram)	None	Yes/Yes/22 days	Favorable
10	Pawar [11]	1st day/F/4000 g/38 w	RT-PCR NA/IgG pos on day 1/IgM neg	YES—maternal RT-PCR positive 3 weeks before delivery	Yes	Hypotension	CRP 1.4, PCT 1.3, ferritin 1500, D-dimer 5088	LV dysfunction (echocardiogram)	IVIG, steroids, inotropes (not specified)	Yes/No/13 days	Favorable
11	Pawar [11]	1st day/M/2020 g/35 w	RT-PCR NA/IgG pos on day 2/IgM neg	YES—asymptomatic, COVID-19 contact 8 weeks before delivery	No	Shock	NT-proBNP >30,000, ferritin 393, D-dimer 5100	Bilateral effusions (CXR); LV dysfunction (echocardiogram)	IVIG, steroids, LMWH	Yes/No/14 days	Favorable
12	Pawar [11]	4 days/F/2000 g/33 w	RT-PCR NA/IgG pos on day 6/IgM neg	YES—asymptomatic, COVID-19 contact 6 weeks before delivery, with positive IgG	No	Severe bradycardia, prolonged QTc and 2:1 AVB; respiratory distress requiring surfactant and MV	ferritin 407, D-dimer 3020	NA	IVIG, steroids	Yes/Yes/16 days	Favorable
13	Pawar [11]	1st day/M/2000 g/36 w	RT-PCR NA/IgG pos on day 1/IgM neg	YES—asymptomatic, COVID-19 contact 6 weeks before delivery	No	Dilated RA/RV, pericardial effusion, RV dysfunction; shock; respiratory distress	CRP 1.2, NT-proBNP >25,000, D-dimer 6848	Pleural effusions (CXR); dilated hypertrophied RV with dysfunction, moderate TR, large thrombus at LPA origin on day 3	IVIG, steroids, inotropes (not specified), alteplase, LMWH	Yes/Yes/19 days	Favorable
14	Pawar [11]	3 days/M/3500 g/38 w	RT-PCR NA/IgG pos on day 5/IgM neg	YES—Febrile illness at 7 months of gestation	No	Hypotension and intermittent bradycardia; respiratory distress requiring MV; feeding intolerance; lethargy	Leukocytosis (18,600), CRP 6.0, PCT 2.1, ferritin 878, D-dimer 6483	NA	IVIG, steroids, inotropes (not specified)	Yes/Yes/13 days	Favorable
15	Pawar [11]	2 days/M/2300 g/34 w	RT-PCR NA/IgG pos on day 12/IgM neg	YES—Febrile illness 2 weeks before delivery	No	Hypotension and intermittent bradycardia; respiratory distress requiring CPAP; rash, pedal edema, oral and skin lesions, skin peeling; feeding intolerance, brown gastric aspirates on day 4, treated like NEC, bleeding; decreased activity on day 2	Thrombocytopenia (39 × 10^9^/L), CRP 2.4, D-dimer 4200	NA	IVIG, steroids, inotropes (not specified), LMWH	Yes/No/38 days	Favorable
16	Pawar [11]	3 days/F/1400 g/34 w	RT-PCR NA/IgG pos on day 1/IgM neg	YES—Asymptomatic, positive RT-PCR at 5th month of gestation	No	Supraventricular tachycardia from day 8; brown gastric aspirates from day 3, frank melena from day 6	CRP 5.0, D-dimer 5100	Bilateral pleural effusions (CXR and echocardiogram)	IVIG, steroids, beta-blockers, LMWH	Yes/No/20 days	Favorable
17	Pawar [11]	2 days/M/1900 g/32 w	RT-PCR NA/IgG pos on day 1/IgM neg	YES—Asymptomatic, positive RT-PCR at 3rd month of gestation	No	Bradycardia with prolonged QTc and 2:1 AVB on day 2; respiratory distress requiring MV	CRP 4.3, D-dimer 6600, IL-6 116	NA	IVIG, steroids, inotropes (not specified), LMWH	Yes/Yes/23 days	Favorable
18	Pawar [11]	2 days/F/1900 g/33 w	RT-PCR NA/IgG pos on day 4/IgM neg	YES—Asymptomatic, COVID-19 contact 8 weeks before delivery	No	Bradycardia with prolonged QTc and 2:1 AVB on day 2	CRP 3.5, D-dimer 10,000	NA	IVIG, steroids, LMWH	Yes/No/18 days	Favorable
19	Pawar [11]	2 days/M/1600 g/33 w	RT-PCR NA/IgG pos on day 5/IgM neg	YES—Asymptomatic, COVID-19 contact 8 weeks before delivery	No	Bradycardia with prolonged QTc and 2:1 AVB on day 4	D-dimer 10,000	NA	IVIG, steroids, LMWH	Yes/No/18 days	Favorable
20	Pawar [11]	4 days/F/2050 g/34 w	RT-PCR NA/IgG pos on day 4/IgM neg	YES—Febrile illness 3 weeks before delivery, with IgG level below cut-off	No	Bradycardia with prolonged QTc and 2:1 AVB on day 4	PCT 1.8, NT-proBNP >25,000, D-dimer 4840	NA	IVIG, steroids, inotropes (not specified), LMWH	Yes/No/11 days	Favorable
21	Pawar [11]	4 days/M/2100 g/34 w	RT-PCR NA/IgG pos on day 4/IgM neg	YES—Febrile illness 3 weeks before delivery, with IgG level below cut-off	No	Bradycardia with prolonged QTc and 2:1 AVB on day 4	PCT 1.4, D-dimer 5932	NA	IVIG, steroids, inotropes (not specified), LMWH	Yes/No/11 days	Favorable
22	Pawar [11]	3 days/F/1000 g/27 w	RT-PCR NA/IgG pos on day 4/IgM neg	YES—Asymptomatic, COVID-19 contact 8 weeks before delivery	No	Bradycardia with prolonged QTc and 2:1 AVB on day 4	PCT 51, NT-proBNP 25,000, D-dimer 10,000	NA	IVIG, steroids, LMWH	Yes/Yes/death on day 11 due to NEC	Death on day 11 (NEC)
23	Pawar [11]	2 days/M/2400 g/36 w	RT-PCR NA/IgG pos on day 6/IgM neg	YES—Asymptomatic, COVID-19 contact 10 weeks before delivery, with positive IgG	No	Cardiogenic shock on day 5; echo-dilated coronaries severe TR, mild MR, ASD, PDA, Severe pulmonary hypertension; refusing feed on day 2	CRP 1.1, D-dimer 4700	Cardiomegaly (CXR); dilated coronaries, severe TR, mild MR, ASD, PDA, PPHN (echocardiogram)	IVIG, steroids, inotropes (not specified), aspirin, LMWH	Yes/Yes/14 days	Favorable
24	Pawar [11]	4 days/M/2000 g/36 w	RT-PCR NA/IgG pos on day 4/IgM neg	YES—Asymptomatic, COVID-19 contact 4 weeks before delivery	No	Shock, bradycardia, mild LV dysfunction; acute renal failure, hyperkalemia; seizures	CRP 1.8, Cr 1.9, K^+^ 6.9, NT-proBNP 14,500, D-dimer 4700	Small ASD, dilated all four chambers, mild LV dysfunction (echocardiogram)	IVIG, steroids, inotropes (not specified), peritoneal dialysis	Yes/Yes/death on day 8 due to MIS-C	Death on day 8 (multi-organ dysfunction)
25	Pawar [11]	1st day/F/2000 g/36 w	RT-PCR NA/IgG pos on day 6/IgM neg	YES—Asymptomatic, COVID-19 contact 4 weeks before delivery, with positive IgG	Yes on day 1	Tachypnea, desaturation; feeding intolerance, vomiting	CRP 6.2, PCT 2.4, NT-proBNP 7361, D-dimer 9734	NA	IVIG, steroids	Yes/No/10 days	Favorable
26	Pawar [11]	2 days/F/1500 g/32 w	RT-PCR NA/IgG neg/IgM neg	YES—Asymptomatic, COVID-19 contact 10 weeks before delivery	No	Bradycardia with prolonged QTc and 2:1 AVB on day 3; respiratory distress	CRP 1.8, D-dimer 12,000	NA	IVIG, steroids, inotropes (not specified)	Yes/No/13 days	Favorable
27	Pawar [11]	2 days/F/1500 g/32 w	RT-PCR NA/IgG below cut-off level/IgM negative	YES—Febrile illness 8 weeks before delivery	No	Bradycardia with prolonged QTc and 2:1 AVB on day 3; respiratory distress	CRP 2.5, NT-proBNP 23,700, D-dimer 10,000	NA	IVIG, steroids	Yes/No/14 days	Favorable
28	Pawar [11]	1st day/M/1900 g/34 w	IgG below cut-off level/IgM negative	YES—Febrile illness 6 weeks before delivery; IgG below cut-off levels, negative IgM	No	Dilated coronaries; not cried after birth; tachypnea, crepitations; pitting edema over chest wall; hepatomegaly	Thrombocytopenia (93 × 10^9^/L), NT-proBNP >25,000, D-dimer 2820	Pleural effusion (CXR); pericardial effusion, dilated coronaries, large PDA, mild TR, normal function on inotropes (echocardiogram); ascites and hepatomegaly (abdomen US)	IVIG, steroids, inotropes (not specified), aspirin, LMWH	Yes/Yes/NA	Favorable
29	Pawar [11]	1st day/F/2700 g/38 w	RT-PCR NA/IgG pos on day 2/IgM neg	YES—Febrile illness 6 weeks before delivery; positive IgG	No	Poor peripheral pulsation and hypotension; not cried after birth; respiratory distress requiring surfactant; mottling	CRP 5.3, NT-proBNP 17,018, D-dimer 3942	Intracardiac thrombus in RA on day 4, normal LV function (echocardiogram)	IVIG, steroids, inotropes (not specified), LMWH	Yes/Yes/NA	Favorable
30	Schoenmakers [38]	At birth/F/1800 g/31 w	RT-PCR neg/Serology neg	YES—maternal SARS-CoV-2 infection during the third trimester associated with a placental inflammatory reaction and subsequent placental dysfunction	No	PPHN; hypoxic respiratory failure managed with surfactant, HFOV and inhaled nitric oxide; elevated creatinine; intraventricular hemorrhage	Leukopenia, thrombocytopenia, elevated liver function tests, elevated Cr, Tn and NT-proBNP, ferritin 14,272, elevated D-dimer	Bilateral opacities (CXR), significantly enlarged LMCA, flattened IVS, mild-to-moderate TR, small PDA with right-to-left shunt (echocardiogram)	IVIG, steroids, inotropes (not specified), aspirin	Yes/Yes/NA	Favorable
31	Shaiba [39]	At birth/F/3004 g/36 w	RT-PCR neg/IgG pos/IgM not tested	YES—The mother tested positive for the SARS-CoV-2 virus in the second trimester, and then again positive 19 days prior to delivery	No	Poor systolic function and hypotension; respiratory distress requiring MV and inhaled nitric oxide; elevated creatinine	CRP 15, PCT 73 (day 1) and 1.22 (day 4), ALT 119 and AST 71, GGT 378, Cr 1, metabolic acidosis, Tn 130, NT-proBNP 3433, ferritin 384, INR 2.59	Moderately dilated LV with poor systolic function, echogenic papillary muscles (could be secondary to ischemia vs. acidosis), wide PDA with a bidirectional shunt and myocarditis (echocardiogram)	IVIG, steroids, plasma transfusion, PGE1, inotropes (dobutamine)	Yes/Yes/12 days	Favorable
32	Shaiba [39]	At birth/F/1700 g/32 w	RT-PCR pos/Serology neg	YES—Ten hours after delivery the mother’s swab result turned out to be positive for SARS-CoV-2	No	Myocarditis, grunting and tachypnea, requiring CPAP; elevated creatinine	CRP 2.02, GGT 141, Tn 51.9, NT-proBNP 5610, ferritin 567, INR 1.06, D-dimer 1060	Bilateral ground glass appearance with bilateral haziness and good lung volume (CXR); myocarditis (echocardiogram)	IVIG, steroids,	Yes/Yes/30 days	Favorable
33	Vivanti [5]	3 days/M/2540 g/35 w	RT-PCR pos/Serology NA	YES—Severe cough since 2 days before hospitalization; positive RT-PCR in blood, amniotic fluid, nasopharyngeal and vaginal swabs	No	Respiratory distress requiring mechanical ventilation; poor feeding on day 3; irritability, axial hypertonia and opisthotonos on day 3	GGT 290, Tn 43	Brain MRI on day 11 showed bilateral gliosis on the deep white periventricular and subcortical matter, with slightly left predominance	None	Yes/Yes/18 days	Favorable

**Abbreviations**: ALT = alanine transaminase, ASD = atrial septal defect; AST = aspartate transaminase, AVB = atrioventricular block; AXR = abdominal X-ray; CPAP = continuous positive airway pressure; Cr = serum creatinine; CRP = C-reactive protein; CT = computed tomography; CXR = chest X-ray; F = female; g = grams; GA = gestational age; GGT = gamma-glutamyl-transferase; HFOV = high frequency oscillator ventilation; IgG = immunoglobulin G; IgM = immunoglobulin M; IL-6 = interleukin-6; INR = International normalized ratio of prothrombin time; IVIG = intravenous immunoglobulin; IVS = interventricular septum; K^+^ = potassium; LAD = left anterior descending coronary artery; LMCA = left main coronary artery; LDH = lactate dehydrogenase; LMWH = low molecular weight heparin; LPA = left pulmonary artery; LV = left ventricle; M = male; MR = mitral regurgitation; MRI = magnetic resonance imaging; MV = mechanical ventilation; NA = not available; NEC = necrotizing enterocolitis; NT-proBNP = N-terminal pro–B-type natriuretic peptide; PCT = procalcitonin; PDA = patent ductus arteriosus; PPHN = persistent pulmonary hypertension of the new-born; QTc = corrected QT interval; RA = right atrium; RCA = right coronary artery; r-TPA = recombinant tissue plasminogen activator; RT- PCR = reverse transcription-polymerase chain reaction; RV = right ventricle; SVT = supraventricular tachycardia; Tn = troponin; TR = tricuspid regurgitation; w = weeks. **Measure units**: Albumin = mg/dl; ALT and AST = U/L; Cr = mg/dl; CRP = mg/dl; D-dimer = mg/dl; Ferritin = ng/mL; GGT = U/L; IL-6 = pg/mL; K^+^ = mEq/L; LDH = U/L; NT-proBNP = pg/mL; PCT = ng/mL; Tn = ng/L.

**Table 3:** Characteristics of neonates and infants under six months of age with MIS-C with fully described cases.

Subject Number	First Author	Age/Sex/Comorbidities	RT-PCR and Serology for SARS-CoV-2	Parental SARS-CoV-2 Exposure	Fever	Organ Involvement	Laboratory Work-Up	Imaging	Treatment	ICU Admission/Need for MV/Length of Hospital Stay	Outcome
1	Acharyya [13]	4 months/M/NA	RT-PCR pos/NA	YES—his mother was also subsequently found positive for SARS-CoV-2.	Yes	Diffuse ectasia of coronary arteries; Erythematous macular rash over the trunk, palm and sole on day 2; Red lips, congested throat and small cervical lymphadenopathy; Irritability	CRP 11.56, Albumin 3, Anemia	Perivascular brightness and diffuse ectasia of coronary arteries (echocardiogram)	IVIG, aspirin	No/No/NA	Favorable
2	Alharbi [47]	1 month/F/No	RT-PCR pos/NA	NA	No	Hypotension, increase in troponin; respiratory distress requiring MV for 12 days	Tn 2410, NT-proBNP 1127, ferritin 6130, IL-6 9.1	Ejection fraction 60%, normal coronary arteries (echocardiogram)	IVIG, Anakinra, inotropes (not specified)	Yes/Yes/13 days	Death
3	Alharbi [47]	3 months/M/No	RT-PCR pos/NA	NA	No	Hypotension, increase in troponin; respiratory distress requiring MV for 15 days	Tn 1294, NT-proBNP 2241, ferritin 813, IL-6 9.3	Ejection fraction 80,5%, normal coronary arteries (echocardiogram)	IVIG, Anakinra	Yes/Yes/73 days	Favorable
4	Cui [16]	55 days/F/No	RT-PCR pos/IgM pos	YES—a week earlier (parents)	No	Tachycardia; productive cough and hypoxic respiratory failure requiring oxygen supplementation through a nasal cannula (day 7 of illness)	CRP 5.6, Tn 25,000, D-dimer 54,000	Ground-glass opacity (CXR); Patchy shadows and ground-glass opacity in the right lung (day 4 of illness), pneumonia (day 9) at chest CT	Inhaled interferon α-1b, reduced glutathione, urso-deoxycholic acid, and traditional Chinese medicine lotus qingwen	No/No/20 days	Favorable
5	Del Barba [17]	38 days/M/No	RT-PCR pos/NA	YES—Both parents diagnosed with COVID-19	Yes	Increase in troponin; rhinitis; modest hypo-reactivity	Thrombocytosis (525 × 10^9^/L), PCT 3.28, Tn 82, NT-proBNP 208, D-dimer 133,000, fibrinogen 128	Mild thickening of broncho-vascular markings, but no pulmonary parenchymal opacities (CXR); minimal pericardial effusion (cardiac MRI)	None	No/No/14 days	Favorable
6	Diggikar [18]	7 days/NA/No	RT-PCR pos/IgG neg/IgM neg	YES—the mother tested positive for SARS-CoV-2 infection one day before the presentation	Yes	Small coronary artery aneurysm; apnoea and respiratory distress requiring MV; reduced tone, sluggish reflexes and seizures	CRP 60, D-dimer 5000	Small coronary artery aneurysm (echocardiogram); diffuse changes involving the periventricular white matter, external capsule and internal capsule, while peripheral bilateral thalami show T2 fluid-attenuated inversion recovery hyperintensity with diffusion restriction (brain MRI)	Steroids, remdesivir, enoxaparin, levetiracetam and phenobarbitone	Yes/Yes/NA	Favorable
7	Dugue [21]	6 weeks/M/No	RT-PCR positive for SARS-CoV-2 and Rhinovirus/NA	NA	Yes	Cough; mottled appearance; brief episodes of upward gaze associated with bilateral leg stiffening and altered responsiveness	Leukopenia (5070)	Excess of temporal sharp transients for age and intermittent vertex delta slowing with normal sleep-wake cycling (long-term EEG)	None	No/No/1 day	Favorable
8	Esteve-Sole [52]	3 months/M/NA	RT-PCR neg/serology neg	YES	Yes	Peripheral extremity changes; gastrointestinal symptoms (not specified); irritability	Leukopenia (4000), CRP 12.8, Albumin 2.3, NT-proBNP 3628, D-dimer 2000	Coronary abnormalities (not specified at echocardiogram)	IVIG, steroids, aspirin, clopidogrel	No/No/NA	Favorable
9	Falah [53]	4 months/M/No	RT-PCR pos/NA	NA	Yes	Isolated coronary artery disease; Rash, conjunctival injection, lips and oral cavity changes; Irritability, neurological symptoms (not specified)	Leukocytosis, CRP 11.56, Albumin 3	Coronary artery dilation (echocardiogram)	IVIG, aspirin	No/No/NA	Favorable
10	Falah [53]	6 months/F/No	RT-PCR pos/NA	NA	Yes	Rash, conjunctival injection, lips and oral cavity changes; Not specified GI symptoms, poor feeding; Irritability, neurological symptoms (not specified)	Leukocytosis, CRP 13.3, Albumin 2.8	Faint opacity in the left mid-lung zone (CXR)	IVIG, aspirin	No/No/NA	Favorable
11	Frauenfelder [22]	32 days (corrected age 37 + 3 weeks)/M/Prematurity	RT-PCR pos/NA	YES—recent contact with family members and asymptomatic healthcare workers	No	Need for inotropes, mildly elevated troponin; significant glottic swelling and copious airway secretions; hypoxic respiratory failure requiring HFOV and inhaled nitric oxide	Lymphopenia (1.45 × 10^9^/L), CRP 4.2, Albumin 2, Tn 138, Ferritin 138, D-dimer 1143	Mild bilateral ground-glass opacities (CXR); Small patent foramen ovale with left-to-right shunt, mild dilation left side structures, and mild MR (echocardiogram)	Steroids, remdesivir, not specified anticoagulation (not specified, because of a line-associated femoral arterial thrombus), inotropes (noradrenaline and adrenaline)	Yes/Yes/18 days	Favorable
12	García-Howard [23]	3 months/F/No	RT-PCR pos/NA	YES	Yes	Rhinorrhea and cough; Diarrhea; Mild hypotonia, staring gaze, clonic movements of the face and right extremities, and repeating sucking movements of the mouth, lasting <5 min	CRP 0.67, Ferritin 385, Increased D-dimer, negative CSF culture	Normal interictal EEG, Normal cerebral MRI	Levetiracetam, Hydroxychloroquine	No/No/9 days	Favorable
13	Giacomet [24]	2 months/F/No	RT-PCR pos/NA	YES— Father and older brother tested positive	Yes	Intermittent tachycardia; mottled skin; non-bloody diarrhea and vomiting	Anemia (Hb 7.9), Tn 103, NT-proBNP 12,507, D-dimer 1918, IL-6 236	Hypokinesia of the inferior left ventricular wall and the inferior interventricular septum, with a mild decrease in the left ventricular ejection fraction	IVIG, Packed red blood cells transfusion	Yes/No/NA	NA
14	Jones [27]	6 months/F/No	RT-PCR pos/NA	YES—her 9-year-old sibling had upper respiratory symptoms 3 weeks before	Yes	Sinus tachycardia during fever; mild congestion and subcostal retractions; Prominent tongue papilla; a blanching, polymorphous, maculopapular rash, limbic sparing conjunctivitis, and dry cracked lips; Irritability	Left-shifted white blood cell count with bandemia, normocytic anemia, CRP 13.3, Albumin 2.8	Faint opacity in the left midlung zone (CXR)	IVIG, aspirin	No/No/NA	Favorable
15	Lad [28]	4 months/F/NA	RT-PCR neg/IgG pos	YES—Father with COVID-19 a month before	Yes	Compensated shock; Vomiting and fresh blood in stool; Lethargy	Neutrophilic leukocytosis; Anemia (Hb 8)	Both coronaries dilated with high z-score > 2.5; Abdominal CT showed dilated jejunum and proximal ileum suggestive of obstruction.	IVIG, steroids, exploratory laparotomy (showing extensively congested ileum and tiny fibrous band about 2.5 cm crossing from antimesenteric border to mesentery in proximal ileum, without any obvious volvulus), packed red blood cells transfusion	No/No/NA	Favorable
16	Luna Santiago [31]	2 months/M/Griscelli syndrome type 2	RT-PCR pos/NA	NA	Yes	Shock; respiratory distress; Hepatosplenomegaly	Pancytopenia (in the context of a familial hemophagocytic lympho-histiocytosis triggered by SARS-CoV-2 at bone marrow aspiration), with increased CRP, D-dimer, triglycerides and IL-6	NA	Steroids, anticoagulation therapy (not specified); inotropes (not specified), multiple transfusions, cyclosporine, cytarabine and inhibitor of JAK signaling	Yes/Yes/NA	Death
17	Malle [41]	6 months/F/Down syndrome and CHD	RT-PCR pos/serology neg	YES—her father had contracted COVID-19	Yes	Heart dysfunction and distributive shock; cough; Maculopapular erythematous rash with peripheral desquamation, edematous hands; Vomiting; Fatigue	Lymphopenia and neutrophilic leukocytosis, increased CRP and PCT, Hypoalbuminemia, Increased Tn, ferritin, D-dimer, fibrinogen and IL-6; Increased STAT3 phosphorylation and increased FcɣRI and ICAM1 on neutrophils and monocytes	Myocarditis and coronary dilatation (echocardiogram)	IVIG, steroids, lopinavir/ritonavir and hydroxychloroquine	Yes/No/4 months	Favorable
18	Mariani [32]	5 months/F/NA	RT-PCR pos/IgG pos	YES—five weeks earlier, her father had tested positive	Yes	Intermittent tachycardia	Thrombocytopenia (36 × 10^9^/L) in the context of a Severe transient pancytopenia with dys-erythropoiesis and dys-megakaryopoiesis; hypoalbuminemia, NT-proBNP 3617, D-dimer 8060	Mild to moderate TR with small pericardial effusion (echocardiogram)	Steroids	No/No/NA	Favorable
19	Marino [42]	5 months/F/No	RT-PCR neg/NA	No	Yes	Isolated coronary artery disease; Erythematous rash at the trunk; Sterile pyuria; Diarrhea; Irritability	CRP 6.4 and PCT 0.96, Tn 13, NT-proBNP 1019, Ferritin 259, D-dimer 1053	Dilatation of both right coronary artery (RCA) and left main coronary artery (LMCA); dim opacity of the left lung base (CXR); gallbladder hydrops (abdominal US);	IVIG, steroids, aspirin	No/No/NA	Favorable
20	Orlanski-Meyer [34]	8 weeks/F/No	RT-PCR neg/IgG pos	YES—both parents tested positive at 2 weeks of age. The positive serology was unlikely to represent the passive transfer of maternal antibodies.	No	Tachycardia; Cracked lips; Profuse watery diarrhea, transient bloody stool, vomiting	Thrombocytosis (958 × 10^9^/L), ALT 173 and AST 140, GGT 274, Albumin 1.4, NT-proBNP 1011, Ferritin 385, Fibrinogen 393, IL-6 37.5	Mild-moderate MR with normal coronary arteries and systolic function (echocardiogram); nonspecific intestinal wall changes and mucosal flattening and splenomegaly (abdominal US); patchy erythema and scattered pinpoint (colonoscopy)	IVIG, steroids, anakinra	No/No/NA	Favorable
21	Rakha [43]	5 months/NA/No	NA/IgG and IgM pos	NA	Yes	Isolated coronary artery disease; not specified respiratory and GI symptoms	Leukocytosis, increased CRP and ferritin	Diffuse ectasia of RCA and LCMA; initial fractional shortening 35% (echocardiogram)	IVIG, aspirin	NA/NA/from 6 to 14 days	Favorable
22	Rakha [43]	5 months/NA/No	NA/IgG and IgM pos	NA	Yes	Isolated coronary artery disease; not specified respiratory and GI symptoms	Leukocytosis, increased CRP and ferritin	Medium aneurysm LMCA, ectasia of LAD and diffuse ectasia of RCA; initial fractional shortening 29% (echocardiogram)	IVIG, aspirin	NA/NA/from 6 to 14 days	Favorable
23	Rakha [43]	3 months/NA/No	NA/IgG and IgM pos	NA	Yes	Isolated coronary artery disease; not specified respiratory and GI symptoms	Leukocytosis, increased CRP and ferritin	Multiple medium and giant aneurysms in left circumflex, and LAD; initial fractional shortening 28% (echocardiogram)	IVIG, aspirin	NA/NA/2 days	Death on second day of admission
24	Rakha [43]	6 months/NA/No	NA/IgG and IgM pos	NA	Yes	Coronary artery disease with myopericarditis; not specified respiratory and GI symptoms	Leukocytosis, increased CRP and ferritin	Ectasia of RCA, LCA, and LAD with decreased contractility; initial fractional shortening 22% (vomiting)	IVIG, aspirin	NA/NA/from 6 to 14 days	Favorable
25	Rakha [43]	36 days/NA/No	NA/IgG and IgM pos	NA	Yes	Supraventricular tachycardia; not specified respiratory and GI symptoms	Leukocytosis, increased CRP and ferritin	Initial fractional shortening 28% (echocardiogram)	None	NA/NA/from 6 to 14 days	Favorable
26	Raut [35]	5 months/M/No	RT-PCR pos/NA	NA	Yes	Isolated coronary artery disease; Skin rash and bilateral non-purulent conjunctivitis; Irritability	CRP 21.5, PCT 8.6, Albumin 2.4, NT-proBNP 2025, Ferritin 975	Normal left ventricular function, with coronary dilatation in LMCA and LAD (echocardiogram)	IVIG, aspirin	No/No/22 days	Favorable
27	Richardson [40]	5 months/F/No	RT-PCR neg/IgG pos a week after hospitalization	NA	Yes	Isolated coronary artery disease; respiratory distress requiring high-flow oxygen on day 5 of illness; Erythematous rash on trunk and extremities (at presentation) with peeling skin on her hands and feet and cracked lips (day 10)	CRP 50, Albumin 2.2, Ferritin 937, D-dimer 6692, Fibrinogen 4700	Coronary artery aneurysm (echocardiogram)	IVIG, steroids, Anakinra then Infliximab, aspirin	Yes/No/NA	Favorable
28	Rodriguez-Gonzalez [36]	6 months/M/short bowel syndrome (secondary to multiple intestinal resections during the neonatal period), antithrombotic prophylaxis due to previous local thrombotic obstructions of the central line	RT-PCR neg/IgG pos on day 21 of illness	NA	Yes	Cardiogenic shock secondary to severe pulmonary hypertension and new onset right ventricular failure; respiratory distress requiring MV	Thrombocytopenia (98 × 10^9^/L), PCT 3.46, Tn 90, NT-proBNP 26,000, Ferritin 7634, Fibrinogen 179, IL-6 198	Massive pulmonary thromboembolism, with a pattern of ground glass and numerous consolidations of predominance in the posterior-basal segments of both lungs (chest CT); Severely dilated right chambers, severe right ventricular systolic dysfunction, and supra-systemic pulmonary hypertension (echocardiogram); irregular pleural line, B-lines, some coalescent, with bilateral patchy distribution, and small peripheral consolidations, which were larger in posterior-basal areas (lung US)	Steroids, Tocilizumab, previous antithrombotic prophylaxis, hydroxychloroquine and inotropic support (milrinone and norepinephrine)	Yes/Yes/21 days	Favorable
29	Saha [37]	25 days/F/Previous hospitalization due to bacterial late-onset sepsis	RT-PCR pos/NA	NO—No family members had signs and symptoms suggestive of SARS-CoV-2. She was in 2 different hospitals previously and may have contracted the virus there.	Yes	Cardiogenic shock; respiratory distress requiring MV; disseminated maculopapular rash; acute kidney injury post-resuscitation; hepatosplenomegaly and greenish watery stool; short-duration seizures	Thrombocytopenia (100 × 10^9^/L), PCR 2.9, Metabolic acidosis, increased NT-proBNP, Ferritin 16,000, D-dimer 16,500	Atelectasis of both lower lobes of lung (chest CT); Significant systolic dysfunction, with ejection fraction of 40% and mild pericardial infusion (echocardiogram)	IVIG, steroids, enoxaparin, inotropes (adrenaline and milrinone), phenobarbitone, furosemide, packed red blood cells transfusion	Yes/Yes/50 days	Favorable
30	Shaiba [44]	30 days/F/NA	RT-PCR pos/NA	NA	Yes	Increased cardiac enzymes; respiratory distress requiring MV; impaired renal function; not specified GI symptoms	Thrombocytopenia (43 × 10^9^/L), CRP 1.1 and PCT 1.7, Hyponatremia (123), Cr 2.44, Tn 684, NT-proBNP 971, Ferritin 2316, D-dimer 5500, Fibrinogen 225,	NA	IVIG, steroids, anakinra, heparin, hydralazine, amlodipine	Yes/Yes/15 days	Death
31	Shaiba [44]	90 days/M/NA	RT-PCR pos/NA	NA	Yes	Increased cardiac enzymes; respiratory distress requiring MV	CRP 5.6, ALT 1070 and 1178, Cr 0.89, Tn 108, NT-proBNP 1370, Ferritin 813, INR 1.49, D-dimer 1320	NA	IVIG, steroids, anakinra, aspirin, enoxaparin, hydralazine, amlodipine, sildenafil	Yes/Yes/75 days	Favorable
32	Villacis-Nunez [45]	4 months/M/Prematurity, twin	RT-PCR pos/IgG pos on day 19 of illness	NA	Yes	Isolated coronary artery disease; respiratory distress requiring non-invasive positive-pressure ventilation; rash, hand and foot swelling, conjunctivitis; diarrhea	Increased CRP (>3)	Giant LAD and RCA aneurysms identified on day 21 of illness (echocardiogram); coronary involvement with possible LAD artery mural thrombus (cardiac CT)	IVIG, steroids, infliximab, remdesivir, aspirin, enoxaparin, clopidogrel	Yes/No/ 26 days	Favorable

**Abbreviations**: ALT = alanine transaminase, ASD = atrial septal defect; AST = aspartate transaminase, AVB = atrioventricular block; AXR = abdominal X-ray; CPAP = continuous positive airway pressure; Cr = serum creatinine; CRP = C-reactive protein; CSF = cerebrospinal fluid; CT = computed tomography; CXR = chest X-ray; EEG = electroencephalogram; F = female; FCɣRI = FCɣ receptor I; g = grams; GGT = gamma-glutamyl-transferase; Hb = Hemoglobin; HFOV = high frequency oscillator ventilation; ICAM1 = Intercellular Adhesion Molecule 1; IgG = immunoglobulin G; IgM = immunoglobulin M; IL-6 = interleukin-6; INR = International normalized ratio of prothrombin time; IVIG = intravenous immunoglobulin; IVS = interventricular septum; K^+^ = potassium; LAD = left anterior descending coronary artery; LMCA = left main coronary artery; LDH = lactate dehydrogenase; LMWH = low molecular weight heparin; LPA = left pulmonary artery; LV = left ventricle; M = male; MR = mitral regurgitation; MRI = magnetic resonance imaging; MV = mechanical ventilation; NA = not available; NEC = necrotizing enterocolitis; NT-proBNP = N-terminal pro–B-type natriuretic peptide; PCT = procalcitonin; PDA = patent ductus arteriosus; PPHN = persistent pulmonary hypertension of the new-born; QTc = corrected QT interval; RA = right atrium; RCA = right coronary artery; r-TPA = recombinant tissue plasminogen activator; RT- PCR = reverse transcription-polymerase chain reaction; RV = right ventricle; STAT3 = Signal Transducer And Activator Of Transcription 3 gene; SVT = supraventricular tachycardia; Tn = troponin; TR = tricuspid regurgitation; US = ultrasound; w = weeks. **Measure units**: Albumin = mg/dl; ALT and AST = U/L; Cr = mg/dl; CRP = mg/dl; D-dimer = mg/dl; Ferritin = ng/mL; fibrinogen = mg/dl; GGT = U/L; Hb = g/dl; IL-6 = pg/mL; K^+^ = mEq/L; LDH = U/L; NT-proBNP = pg/mL; PCT = ng/mL; Tn = ng/L.

**Table 4:** Characteristics of infants with MIS-N and MIS-C belonging to cohort studies and not fully described.

First Author	Total Number of Children with MIS-C	Number of Infants under Six Months of Age	Available Description
Abdel-Haq [46]	13	1	A 3-months-old girl with a positive RT-PCR for SARS-CoV-2 and dilated coronary arteries, successfully treated with IVIG and infliximab
Alharbi [47]	5	2	Full description in Table 3 (Case 2 and Case 3)
Antúnez-Montes [48]	95	3	3 infants under the age of a month, but their specific clinical characteristics were not described
Caro-Domínguez [49]	37	1	A 6-months-old boy with cardiac failure and positive RT-PCR for SARS-CoV-2
Chandran [50]	17	3	Two infants survived (a 1-month-old infant who had prematurity as comorbidity and a 6-months-old infant who had previously undergone Kasai procedure at 2 months of age for biliary atresia). A 1-month-old infant with refractory thrombocytopenia and multiorgan involvement, treated with IVIG, methylprednisolone and cyclosporine, died.
Dufort [51]	191	1	A neonate with MIS-N, born to a positive mother (asymptomatic at delivery) who presented with fever and left breast cellulitis between 14 and 28 days of age. Laboratory work-up showed increasing troponin levels; echocardiogram showed good ventricular function and unremarkable coronary arteries. Two molecular tests for SARS-CoV-2 were negative. The discharge diagnoses were cellulitis, myocarditis, and shock.
Godfred-Cato [54]	85	1	The specific clinical characteristics of infants < 6 months of age are not described.
Grewal [55]	92	9	Nine infants under the age of 6 months, none with acute kidney injury.
Güllü [56]	320	3	A 1-month-old girl, followed up with aortic coarctation, resulted to be positive for SARS-CoV-2 and had fever and poor feeding. A 3-months-old boy, followed up with ventricular septal defect and pulmonary hypertension, had fever, diarrhoea (leading to a severe dehydration and lack of urine). A 6-days-old boy, who applied with fever, vomiting, decreased feeding and respiratory distress for 2 days. It was learned that his aunt and grandfather had been diagnosed with COVID-19 and they had loved and cared for the baby.
Mehra [57]	120	1	Among four deaths, one was a 3-month-old infant with acute COVID-19 related severe acute respiratory distress syndrome and shock
Niño-Taravilla [58]	26	2	Two neonates with MIS-C (the specific clinical characteristics are not described)
Shaiba LA [44]	36	2	Full description in Table 3 (Case 30 and Case 31)

## Data Availability

The data presented in this study are available in the included articles. No new data were created or analyzed in this study.
